# Long-term reoperation rate following primary ventral hernia repair: a register-based study

**DOI:** 10.1007/s10029-022-02645-3

**Published:** 2022-07-08

**Authors:** A Katawazai, G Wallin, G Sandblom

**Affiliations:** 1 Departments of Surgery, School of Medical Sciences, Örebro University Hospital, Örebro University, Stockholm, Sweden; 2 Örebro University Hospital, Faculty of Medicine and Health, Örebro University, Stockholm, Sweden; 3 Department of Clinical Science and Education Södersjukhuset, Karolinska Institute, Stockholm, Sweden; 4 Department of Surgery, Karlskoga Hospital, 691 44 Karlskoga, Sweden; 5 Department of Surgery, Södersjukhuset, Stockholm, Sweden

**Keywords:** Primary ventral hernia, Umbilical hernia, Epigastric hernia, Hernia repair, Recurrence, Reoperation

## Abstract

**Background:**

The aim of this study was to analyse the risk for reoperation following primary ventral hernia repair.

**Methods:**

The study was based on umbilical hernia and epigastric hernia repairs registered in the population-based Swedish National Patient Register (NPR) 2010–2019. Reoperation was defined as repeat repair after primary repair.

**Results:**

Altogether 29,360 umbilical hernia repairs and 6514 epigastric hernia repairs were identified. There were 624 reoperations registered following primary umbilical repair and 137 following primary epigastric repairs. In multivariable Cox proportional hazard analysis, the hazard ratio (HR) for reoperation was 0.292 (95% confidence interval (CI) 0.109–0.782) after open onlay mesh repair, 0.484 (CI 0.366–0.641) after open interstitial mesh repair, 0.382 (CI 0.238–0.613) after open sublay mesh repair, 0.453 (CI 0.169–1.212) after open intraperitoneal onlay mesh repair, 1.004 (CI 0.688–1.464) after laparoscopic repair, and 0.940 (CI 0.502–1.759) after other techniques, when compared to open suture repair as reference method. Following umbilical hernia repair, the risk for reoperation was also significantly higher for patients aged < 50 years (HR 1.669, CI 1.389–2.005), for women (HR 1.401, CI 1.186–1.655), and for patients with liver cirrhosis (HR 2.544, CI 1.049–6.170). For patients undergoing epigastric hernia repair, the only significant risk factor for reoperation was age < 50 years (HR 2.046, CI 1.337–3.130).

**Conclusions:**

All types of open mesh repair were associated with lower reoperation rates than open suture repair and laparoscopic repair. Female sex, young age and liver cirrhosis were risk factors for reoperation due to hernia recurrence, regardless of method.

## Introduction

Primary ventral hernia (PVH) in the linea alba is one of the most common conditions requiring surgical management. Most of these hernias present in or above the umbilicus (epigastric hernia). PVH should be distinguished from incisional hernia which may also develop in the midline but only after a previous surgical procedure. PVH is often asymptomatic, but can present with symptoms such as discomfort, pain, or acute incarceration. Some surgeons advocate repair of an asymptomatic hernia, while others recommend watchful waiting. While evidence supporting watchful waiting for patients with asymptomatic umbilical and epigastric hernias is weak, guidelines do recommend this strategy [1]. Each year approximately 4500 PVHs are surgically repaired in Sweden [2]. The prevalence of PVH among the general adult population, however, is estimated to be less than 1% [3]. Although many surgeons consider PVH repair a relatively simple and straightforward procedure, many technical issues warrant careful consideration when performing the repair. PVH repair is a challenging procedure because of heterogeneity of hernia presentation, truncal obesity, uncertainty regarding the safety of various methods of repair, and risk for rare but potentially serious complications. A high long-term recurrence rate is widely considered the most important complication following PVH repair. According to previous research, postoperative complications such as haematoma, seroma, SSI, and pain are factors that increase the risk for developing recurrence [4–6]. The best surgical approach for repair of PVH remains controversial, and the method chosen usually depends on the surgeon’s preference and competence. Suture versus mesh, type of mesh, and different approaches for mesh placement are issues that are a continuous topic of debate. There is strong evidence that the use of mesh for open umbilical or epigastric hernia repair reduces the rate of recurrence for large hernia defects, but evidence is lacking for defect sizes less than 1 cm [7, 8]. Layer of mesh placement is another issue that is poorly investigated. Another controversial issue is PVH repair in patients with comorbidity, in particular truncal obesity. Some studies suggest that the laparoscopic approach may be better for patients with a large hernia or high risk for wound complications [9–12]. Ideally the surgical approach should be tailored to the characteristics of the individual patient, and the decision should be shared.

This large retrospective register study was designed to examine the long-term recurrence rate after different surgical approaches for PVH repair. The aim was not only to evaluate surgical approach, but also to identify patient groups with higher risk for reoperation for hernia recurrence, and to explore the impact of sex, age and comorbidity as independent risk factors for reoperation.

## Methods

The study was based on data retrieved from the Swedish National Patient Register (NPR). The NPR is a national register that covers all medical care, private as well as public [13]. At each outpatient visit or inpatient care discharge, the responsible physician registers all relevant diagnoses according to ICD codes and Nordic Medico-Statistical Committee (NOMESCO) Classification of Surgical Procedure codes [14]. Since 1987, the NPR has included all in-patient care in Sweden, and since 2001, the register has also covered outpatient visits to a doctor, including day-case surgery. The validity of the NPR has been shown to be high for the diagnoses relevant to the present study [15].

All patients undergoing surgery with any of the NOMESCO Classification of Surgical Procedure codes JADNN (umbilical hernia repair) or JAENN (epigastric hernia repair) 2010–2019 were identified. The register enabled distinguishing between 7 methods of repairs for umbilical hernias; open suture repair, open onlay mesh repair, open interstitial mesh repair, open sublay mesh repair, open intraperitoneal onlay mesh (IPOM)repair, laparoscopic repair and “other repair” or method unknown. Epigastric hernias were categorized as suture repair, mesh repair or other method of repair.

The study cohort was defined by the first discharge note or outpatient visit with any of these codes. All patients in the cohort were followed until December 31, 2019, or until the patient died or underwent surgery with the same NOMESCO surgical procedure code. Reoperations with the same NOMESCO procedure code was assumed to have been carried out for a recurrence at the same location. As the NPR has a national coverage and the Swedish Personal Registration numbers unique for each Swedish resident are used at all units reports to the NPR, we could trace each individual from the primary repair until the reoperation, regardless of where the reoperation was carried out. ICD codes for comorbidity present prior to hernia repair were also identified as follows: K703, K742, K743, K744 or K745 (liver cirrhosis), E10-E14 (diabetes mellitus), E66 (obesity), and J44 (chronic obstructive pulmonary disease). Patients with a history of any of these diagnoses were considered to have them in chronic form. Obesity was assigned by the responsible physician based on clinical relevance, without national criteria.

The study was approved by the Swedish Ethical Review Authority (2020–04,429). Registration in the National Patient Register is mandatory, but no data that could be traced to an individual was retrieved from the register, we could thus not ask for consent.

### Statistical methods

Time to event analyses were carried out, with time from the first registered repair defined as index repair and the first occurring repair with the same anatomical location considered as reoperation. Procedure codes registered within 30 days after the first repair were not included as they may have referred to identical procedures coded by two different care providers. Cox proportional hazard analyses were carried out, with method of repair, age, sex, and comorbidity as covariates.

## Results

**Fig. 1:**
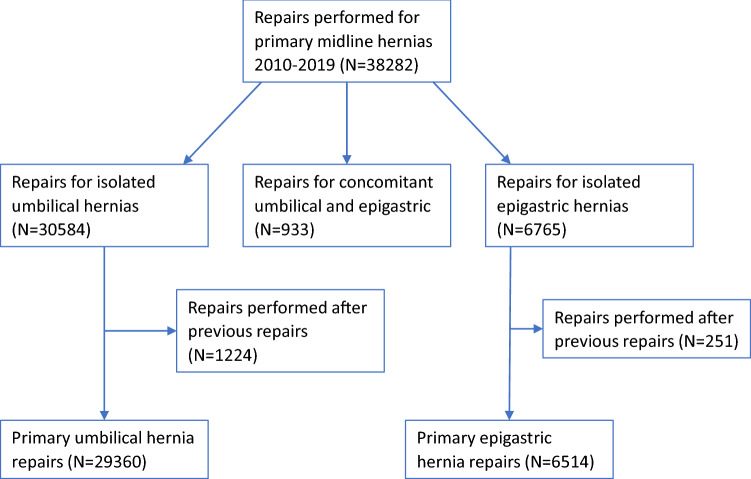
Cohort assembly

The total number of PVHs during the study period was 38 282. After exclusion of procedures carried out on recurrent hernias, 35 874 procedures remained for the study cohort, 29,360 procedures for primary umbilical hernia and 6 514 procedures for epigastric hernia (Fig. 1). Median age was 49 years (interquartile range, IQR, 38–61 years), men being older than women (53 years, IQR 43–64 years, vs 41 years, IQR 33–54 years). Comorbidities were registered in 13.6%, the most common being diabetes mellitus and obesity (5.8 and 5.3%, respectively).

Umbilical hernia repair was more commonly performed in men than women (18,754 versus 10,606) while epigastric hernia repair was more common in women than men (3752 versus 2762) (Table 1).

**Table 1:** Baseline characteristics. As some patients underwent umbilical hernia repair as well as epigastric hernia repair, the entire cohort is smaller than the sum of other two cohorts

	Entire cohort (*N* = 35,874)	Patients undergoing umbilical hernia repair (*N* = 29,360)	Patients undergoing epigastric hernia repair (*N* = 6514)
Sex			
Men	21 822 (59.8%)	18,754 (63.9%)	2762 (42.4%)
Women	14 676 (40.2%)	10,606 (36.1%)	3752 (57.6%)
Median age all patients, years (quartiles)	49 (38–61)	50 (39–62)	45 (33–58)
Median age women, years (quartiles)	41 (33–54)	41 (33–53)	44 (34–56)
Median age men, years (quartiles)	53 (43–64)	54 (44–64)	46 (32–59)
Comorbidities			
Liver cirrhosis	197 (0.5%)	179 (0.6%)	16 (0.2%)
Diabetes	2132 (5.8%)	1953 (6.7%)	163 (2.5%)
Obesity	1927 (5.3%)	1719 (5.9%)	177 (2.7%)
Chronic Obstructive Pulmonary Disease	733 (2.0%)	612 (2.1%)	107 (1.6%)

The most common repair method for both umbilical and epigastric hernia was open suture repair (*N* = 19,391 and *N* = 4835 resp.). Open interstitial mesh was the second most commonly used for umbilical hernia (*N* = 4363), and open sublay mesh repair the third most commonly used technique (*N* = 2604). The median age of patients undergoing umbilical or epigastric hernia repair was 49 years (Table 2).

**Table 2:** Univariable and multivariable Cox proportional hazard analysis of risk for reoperation following epigastric hernia repair ((*N* = 6514)

	Univariable analysis		Multivariable analysis	
	Hazard ratio (95% confidence interval)	*p*	Hazard ratio (95% confidence interval)	*p*
Method or repair (reference suture repair, *N* = 4835)				
Mesh repair (*N* = 1620)	0.773 (0.487–1.228)	0.275	0.887 (0.555–1.418)	0.617
Other repair (*N* = 59)	1.073 (0.150–7.692)	0.944	1.136 (0.158–8.147)	0.899
Age < median (50 years, *N* = 3917)	2.129 (1.405–3.225)	< 0.001	2.046 (1.337–3.130)	< 0.001
Women (*N* = 3752)	1.055 (0.738–1.507)	0.769	0.989 (0.691–1.414)	0.950
Chronic obstructive pulmonary disease (*N* = 107)	1.091 (0.270–4.411)	0.903	1.727 (0.417–7.147)	0.451
Liver cirrhosis (*N* = 16)	–	–	–	–
Diabetes (*N* = 163)	–	–	–	–
Obesity (*N* = 177)	0.641 (0.158–2.592)	0.533	0.798 (0.196–3.242)	0.752

**Fig. 2:**
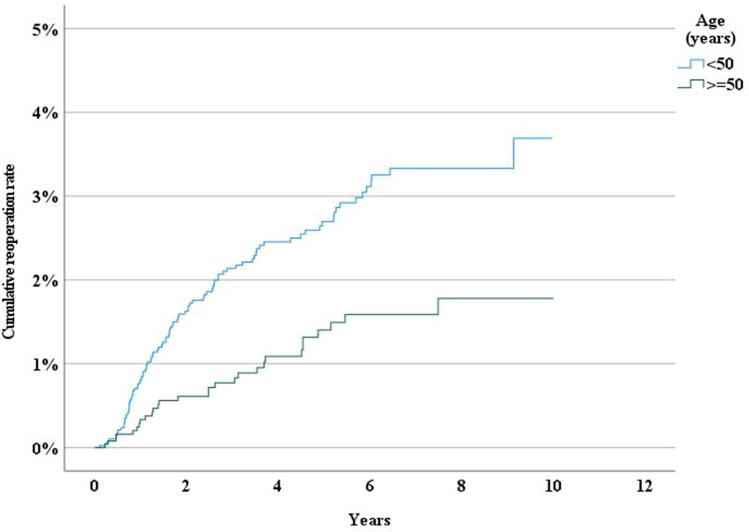
Incidence rate of reoperation following epigastric hernia repair by age

**Fig. 3:**
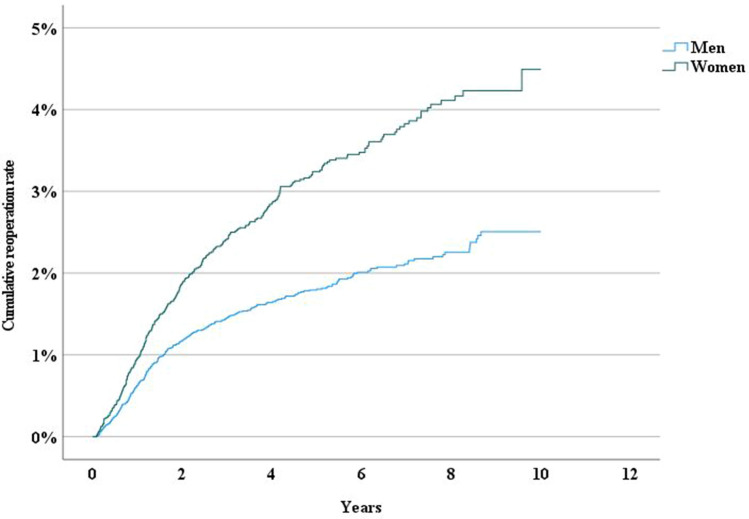
Incidence rate of reoperation following umbilical hernia repair by sex

Age and sex were independent risk factors for reoperation. Patients younger than the median (< 50 years) had a higher risk for reoperation following both umbilical and epigastric hernia repair. (Figs. 2 and 3). We also found that women had a significantly higher risk for reoperation than men (*P* < 0.001) (Fig. 3).

**Fig. 4:**
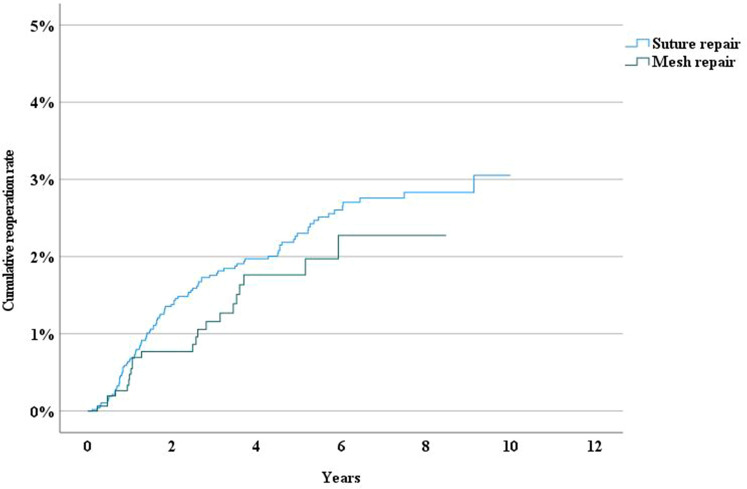
Incidence rate of reoperation following epigastric hernia repair by method of repair

**Fig. 5:**
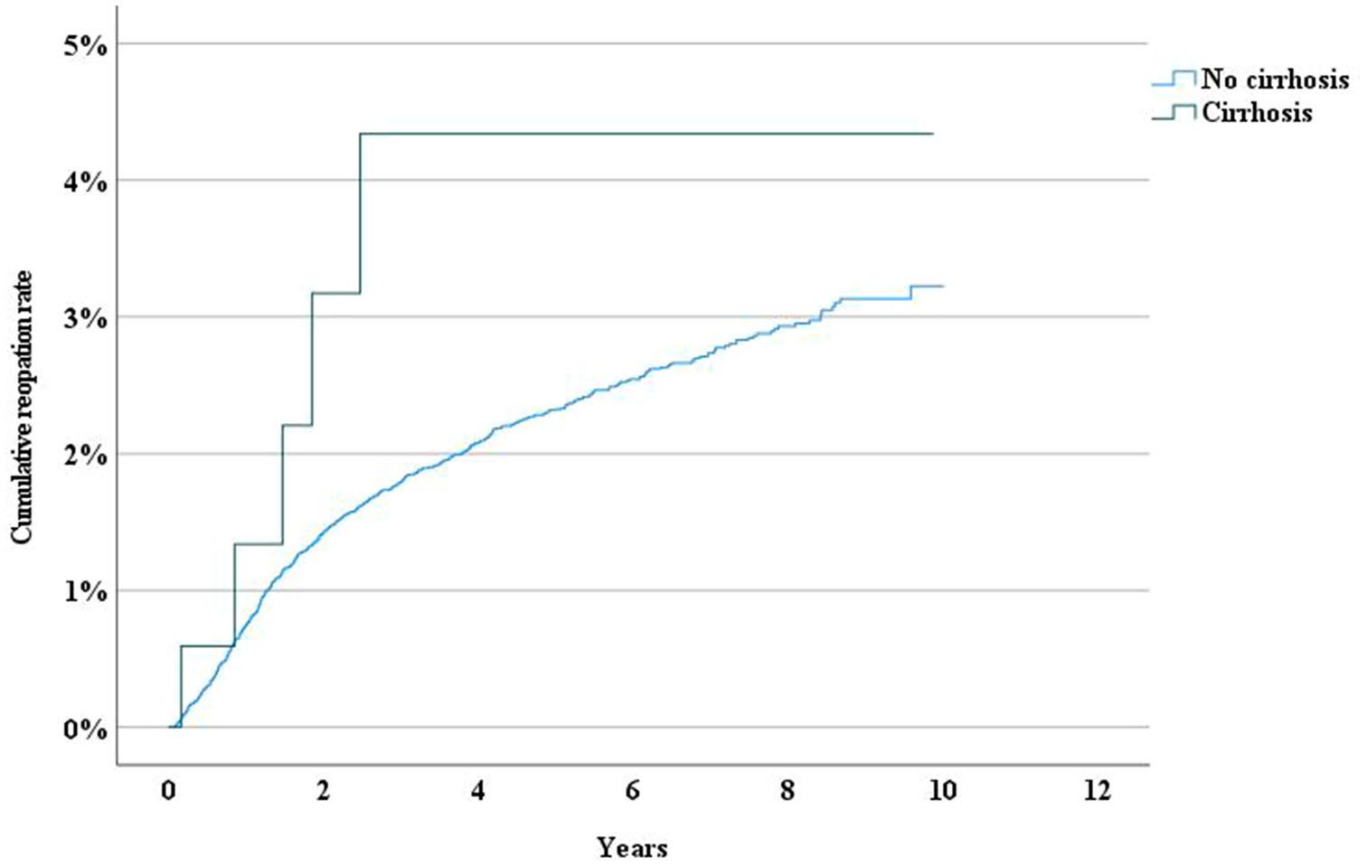
Incidence rate of reoperation following umbilical hernia repair by history of liver cirrhosis

**Fig. 6:**
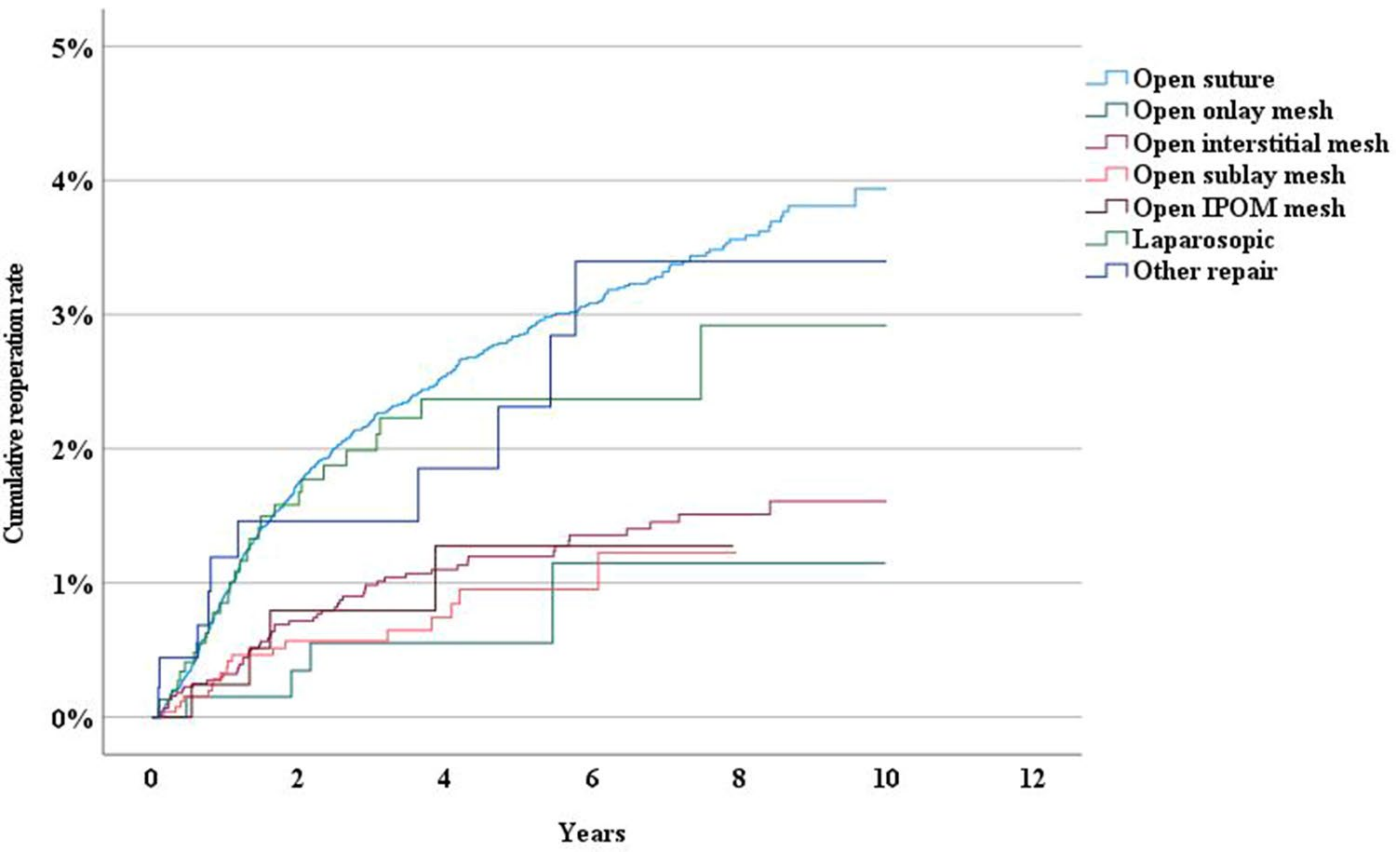
Incidence rate of reoperation following umbilical hernia repair by method of repair

The long-term reoperation rate following epigastrical hernia repair was higher after suture repair than after mesh repair (Fig. 4). The reoperation rate was higher after open suture repair, laparoscopic repair, and “other repair” for umbilical hernias, whereas open IPOM-repair, open sublay, interstitial mesh placement, and onlay mesh repair had lower reoperation rates due to recurrence (Fig. 5). Patients with liver cirrhosis had a significantly higher risk for reoperation compared to those without (HR 2.544, 95% CI 1.049–6.170)(Fig. 6).

Compared to previous reports [12, 16, 17] laparoscopic repair in this study had a higer recurrence rate (Fig. 5).

**Fig. 7:**
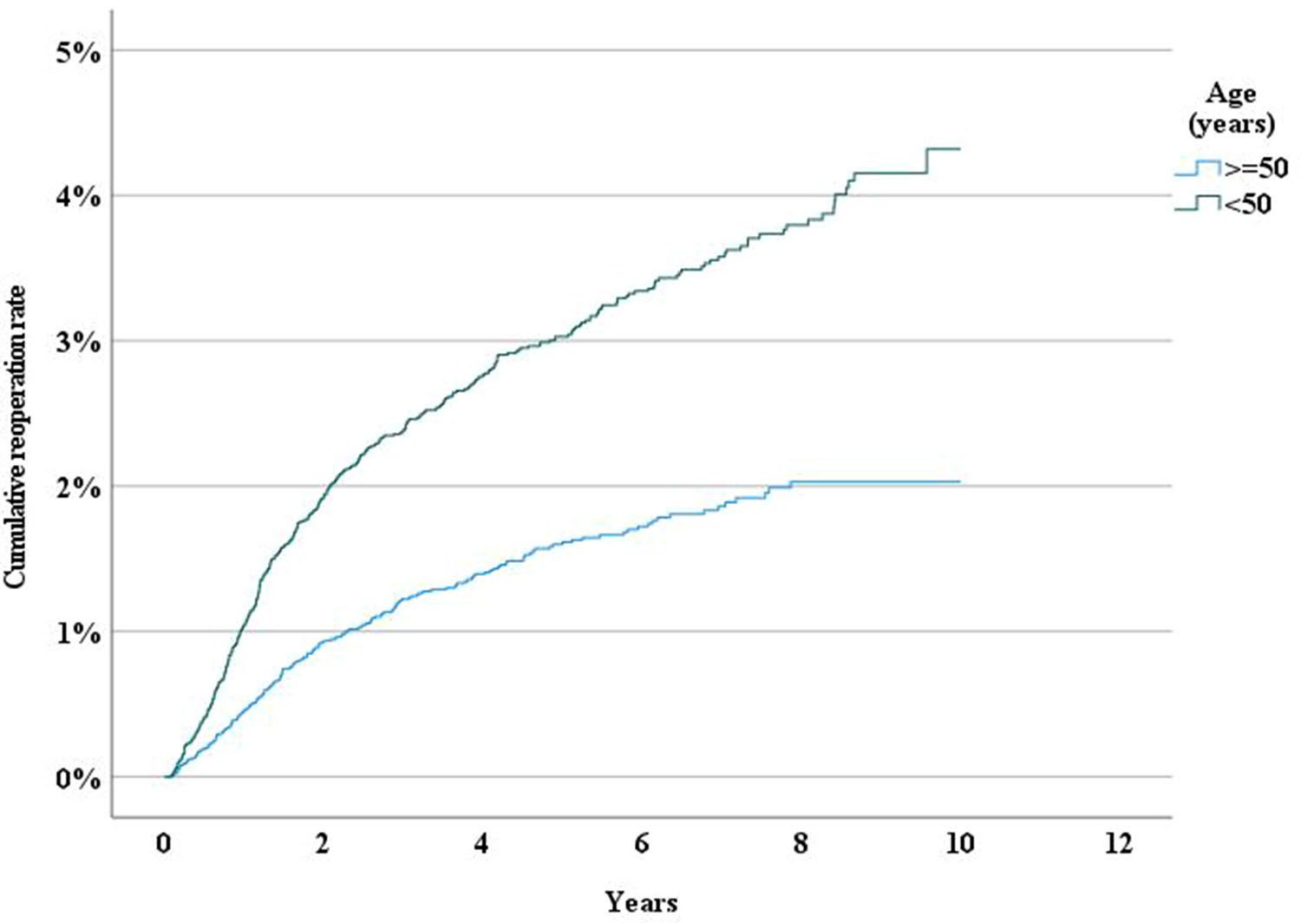
Incidence rate of reoperation following umbilical hernia repair by age

The reoperation rate was not significantly higher in patients with a history of chronic obstructive pulmonary disease (P0.937), diabetes (P0.896), or obesity (P0.521)(Fig. 7).

## Discussion

This nationwide population-based study follows reoperation for recurrence after repairs for umbilical and epigastric hernia as practiced in the community at large over the past 10 years. Although recurrence rates in general are low, the present study shows that mesh reinforcement should be used in selected cases based on the risk factors identified in this study.

In most cases, umbilical and epigastric hernias can be safely repaired using a synthetic polypropylene mesh as reinforcement [1]. Five anatomical layers for mesh placement are possible: onlay, inlay or interstitial, sublay, preperitoneal, and intraperitoneal onlay mesh (IPOM). Few studies have compared outcomes after using these mesh locations. Guidelines usually recommend sublay mesh repair [1]. According to the European Hernia Society guidelines, there is no strong evidence in favour of any particular method of repair for small PVH (defect size < 1 cm). However, a systemic review-guided consensus of experts advocated sublay mesh repair for hernias with defect size > 1 cm [7]. In the present study, open IPOM mesh repair had a better outcome than laparoscopic repair. The recurrence rates following laparoscopic repair may, however, be explained by selection bias since there were probably larger hernias in this group. Large hernia defects and hernias in patients with higher risk for surgical site infection (SSI) are usually repaired laparoscopically [6, 9, 11]. However, the effectiveness of laparoscopic ventral hernia repair (LVHR) may be limited, especially in defects size greater than 80 cm^2^, with a higher recurrence rate, which some physicians may consider as a contraindication [18]. Unfortunately, the register lacks data on hernia defect size and other anatomical data.

Most previous studies have focused on repair materials and methods, while other important risk factors for recurrence or complications such as age, gender, obesity, and comorbidity are less studied. These should be taken in consideration when choosing the method of repair for recurrence.

Women had a 40% higher risk for reoperation after repair of umbilical hernias (Fig. 3), even when adjusting for method of repair (Table 3). This could be explained by distension of the linea alba following one or more pregnancies. Usually there is some degree of weakness in the linea alba or rectus abdominis diastasis, both of which may increase the risk for recurrence following hernia repair [19] Some studies recommend mesh in patients with concomitant rectus muscle diastasis[19–21], although evidence is weak. More research is needed to explore why women are at higher risk for reoperation. Hypothetically, postpartum rectus abdominis diastasis is a risk factor [12, 20, 22].

**Table 3:** Univariable and multivariable Cox proportional hazard analysis of risk for reoperation following umbilical hernia repair ((*N* = 29,360)

	Univariable analysis		Multivariable analysis	
	Hazard ratio (95% confidence interval)	*p*	Hazard ratio (95% confidence interval)	*p*
Method or repair (reference category open suture repair, *N* = 19 391)				
Open onlay mesh (*N* = 703)	0.264 (0.099–0.705)	0.008	0.292 (0.109–0.782)	0.014
Open interstitial mesh (*N* = 4363)	0.431 (0.326–0.569)	< 0.001	0.484 (0.366–0.641)	< 0.001
Open sublay mesh (*N* = 2604)	0.334 (0.209–0.535)	< 0.001	0.382 (0.238–0.613)	< 0.001
Open IPOM mesh (*N* = 449)	0.412 (0.154–1.101)	0.077	0.453 (0.169–1.212)	0.115
Laparoscopic repair (*N* = 1400)	0.926 (0.636–1.347)	0.687	1.004 (0.688–1.464)	0.710
Other repair (*N* = 450)	0.915 (0.490–1.712)	0.782	0.940 (0.502–1.759)	0.846
Age < median (50 years, *N* = 14 430)	2.006 (1.694–2.374)	< 0.001	1.669 (1.389–2.005)	< 0.001
Women (*N* = 10 606)	1.756 (1.499–2.056)	< 0.001	1.401 (1.186–1.655)	< 0.001
Chronic obstructive pulmonary disease (*N* = 612)	0.688 (0.343–1.382)	0.294	0.972 (0.480–1.969)	0.937
Liver cirrhosis (*N* = 179)	1.869 (0.775–4.508)	0.164	2.544 (1.049–6.170)	0.039
Diabetes (*N* = 1953)	0.674 (0.455–0.998)	0.049	0.896 (0.595–1.350)	0.896
Obesity (*N* = 1719)	1.032 (0.730–1.458)	0.860	1.123 (0.788–1.602)	0.521

More research is also needed to explain why younger patients are at higher risk for reoperation for recurrence. One possible explanation for this is the greater chance of being reoperated for recurrence with mild or moderate symptoms in younger patients, whereas a recurrence in the elderly is less likely to be reoperated [23] Reoperation for recurrence is, in this context, a surrogate measure that may be biased by age. Elderly and patients with high comorbidity that develop recurrence following repair of a ventral hernia are often treated conservatively and are hence not registered to have undergone repair of a recurrence. Younger patients on the other hand are physically more active and dependent of fully functional abdominal wall, which also could be a possible reason for reoperation due to recurrence.

Recurrence rate is the most frequent outcome studied, while data on patient-reported outcomes are generally lacking [7]. Another important issue that needs further research is chronic pain after different surgical approaches for PVH repair.

A weakness of the present study is that “other repairs” are not defined. This group may include suture repairs as well as mesh repairs. On the other hand, there were only a few procedures categorized as “other repair”. However, in order to cover all repairs used, other repair was included as a separate category in the analyses. Another weakness is that data on type of laparoscopic approach and modifications performed, and size of hernia were not available. Size of hernia defect is another important issue that plays an important role in the surgeon’s decision on method of repair.

The prevalence of co-morbidities was lower than expected in this patient group. This may be explained by incomplete coverage of the NPR. Obesity is very common in patients with umbilical hernias, but the ICD code of obesity (E66) is only assigned to a patient in case it warrants specific treatment or intervention. There may thus be a great underreporting of this ICD code.

## Conclusion

In this nationwide population-based register study on repair of umbilical and epigastric hernias, women were found to have higher reoperation rate than men. Another group with a higher for reoperation risk was individuals younger than 50 years old. Both groups had a significantly higher risk for reoperation due to recurrence, regardless of primary method of repair. Regarding comorbidities, patients with liver cirrhosis had a higher recurrence rate regardless of type of primary repair.

The results of this study suggest that all forms of open mesh repair (sublay, interstitial, onlay, and open IPOM) generally have a better outcome than the other three methods (open suture, laparoscopic repair, and other/unknown repair). Open onlay, open interstitial and open sublay mesh repairs had significantly better outcome than suture repair. Of the open mesh repairs, onlay mesh repair had the lowest risk for reoperation.
